# Gene Selection and Evolutionary Modeling Affect Phylogenomic Inference of Neuropterida Based on Transcriptome Data

**DOI:** 10.3390/ijms20051072

**Published:** 2019-03-01

**Authors:** Yuyu Wang, Xiaofan Zhou, Liming Wang, Xingyue Liu, Ding Yang, Antonis Rokas

**Affiliations:** 1 College of Plant Protection, Hebei Agricultural University, Baoding 071001, China; wangyy_amy@126.com (Y.W.); wanglm1990@126.com (L.W.); 2 Department of Entomology, China Agricultural University, Beijing 100193, China; 3 Department of Biological Sciences, Vanderbilt University, Nashville, TN 37235, USA; zhouxiaofan1983@163.com; 4 Guangdong Province Key Laboratory of Microbial Signals and Disease Control, Integrative Microbiology Research Centre, South China Agricultural University, Guangzhou 510642, China

**Keywords:** transcriptome, phylogenomics, site-heterogeneous model, Neuropterida

## Abstract

Neuropterida is a super order of Holometabola that consists of the orders Megaloptera (dobsonflies, fishflies, and alderflies), Neuroptera (lacewings) and Raphidioptera (snakeflies). Several proposed higher-level relationships within Neuropterida, such as the relationships between the orders or between the families, have been extensively debated. To further understand the evolutionary history of Neuropterida, we conducted phylogenomic analyses of all 13 published transcriptomes of the neuropterid species, as well as of a new transcriptome of the fishfly species *Ctenochauliodes similis* of Liu and Yang, 2006 (Megaloptera: Corydalidae: Chauliodinae) that we sequenced. Our phylogenomic data matrix contained 1392 ortholog genes from 22 holometabolan species representing six families from Neuroptera, two families from Raphidioptera, and two families from Megaloptera as the ingroup taxa, and nine orders of Holometabola as outgroups. Phylogenetic reconstruction was performed using both concatenation and coalescent-based approaches under a site-homogeneous model as well as under a site-heterogeneous model. Surprisingly, analyses using the site-homogeneous model strongly supported a paraphyletic Neuroptera, with Coniopterygidae assigned as the sister group of all other Neuropterida. In contrast, analyses using the site-heterogeneous model recovered Neuroptera as monophyletic. The monophyly of Neuroptera was also recovered in concatenation and coalescent-based analyses using genes with stronger phylogenetic signals [i.e., higher average bootstrap support (ABS) values and higher relative tree certainty including all conflicting bipartitions (RTCA) values] under the site-homogeneous model. The present study illustrated how both data selection and model selection influence phylogenomic analyses of large-scale data matrices comprehensively.

## 1. Introduction

Neuropterida is a super order of Holometabola that is composed of the orders Neuroptera (lacewings), Megaloptera (dobsonflies, fishflies and alderflies), and Raphidioptera (snakeflies). Neuropterids are generally delicate insects that have two pairs of membranous wings with highly reticulate venation. Phylogenetically, neuropterids are thought to be the sister group of the Coleoptera + Strepsiptera clade [1,2]. Extant Neuropterida comprises ca. 6500 species in 20 families [3], although their fossil records are extremely rich, with many (now extinct) families present during the Late Paleozoic and throughout the Mesozoic. Due to their generalized adult morphology as well as their tremendous disparity in larval morphology and life style, there are numerous competing hypotheses concerning the ordinal and family-level relationships within Neuropterida.

Recent studies focused on the higher-level phylogeny of Neuropterida corroborate the sister group relationship between Megaloptera and Neuroptera based on both morphological [4,5,6] and molecular data [7,8], including mitogenomic [9,10], transcriptomic [2], and genomic data [11]. Currently, the largest uncertainty concerns the interfamilial phylogeny of Neuroptera, particularly with respect to the phylogenetic positions of Nevrorthidae, Coniopterygidae, and some other families previously placed in the suborder of Hemerobiiformia [4]. For instance, Nevrorthidae, which has an exclusive aquatic larval lifestyle, was considered to be the sister-group of all other lacewing families and stood as an independent suborder Nevrorthiformia [4]. A more recent analysis, however, recovered Nevrorthidae to be the sister-group of Sisyridae, another lacewing family with aquatic larvae [12]. Similarly, Coniopterygidae, whose members are commonly called dusty-wings, have their bodies covered with secreted wax and also exhibit strongly reduced wing venation, and were thought to be the sister-group to the rest of Neuroptera, as first proposed by Withycombe [13] and later supported in a molecular phylogenetic analysis by Winterton et al. [12]. However, other analyses have placed this family in more derived positions, either close to Sisyridae or close to families such as Dilaridae and Mantispidae [4,5,7,14,15,16,17,18].

In recent years, high-throughput transcriptome sequencing (RNA-seq) has greatly augmented the collection of orthologous sequence data for phylogenomic studies [2,19,20,21]. Up to now, there are 13 published transcriptomes of Neuropterida, representing all of Neuroptera, Megaloptera, and Raphidioptera, as well as most major lineages within these three orders, although genome-scale data set for fishflies (Chauliodinae; Megaloptera) still remained absent. At the same time, novel methods of phylogenomic inference (coalescent-based inference [22,23,24]), models of sequence evolution (e.g., site-heterogeneous model [25,26]) and measures of conflict among phylogenetic trees (e.g., internode certainty (IC) and related measures [27,28]) have greatly aided the inference and evaluation of relationships from phylogenomic data.

In this study, we combine the power of RNA-seq data with recently developed methods of phylogenetic inference to reconstruct and evaluate the higher-level phylogeny of Neuropterida. We newly sequenced and analyzed the transcriptome of the fishfly species *Ctenochauliodes similis* Liu and Yang, 2006 (first transcriptome of the subfamily Chauliodinae, a major lineage of Megaloptera), and used it together with the transcriptomes of 21 other holometabolan species, including 13 publicly available transcriptomes of Neuropterida, to reconstruct the phylogeny of this super order based on phylogenomic analyses of a 1392 gene data matrix.

## 2. Results

### 2.1. Illumina Sequencing, Sequence Assembly, and Data Matrix Construction

**Figure 1:**
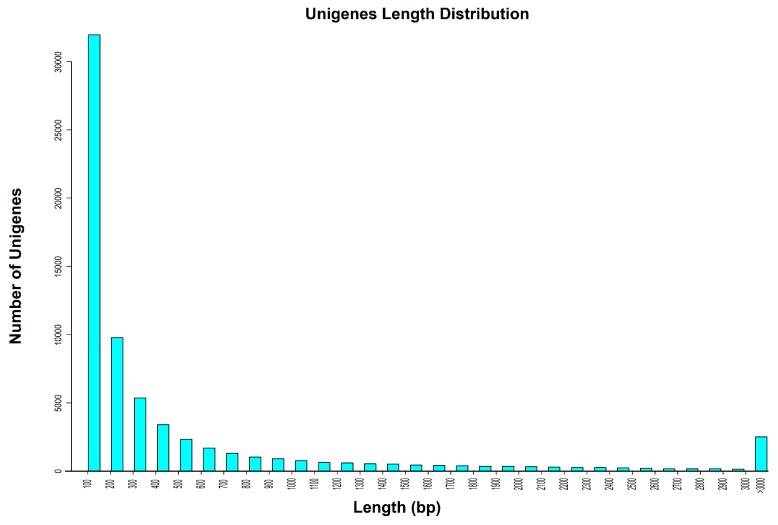
Sequence-length distribution of uni-genes. The *x*-axis represents the length range, the *Y*-axis is the number of uni-genes.

Illumina sequencing of the transcriptome of *C. similis* (see Materials and Methods) yielded a total of 26,988,698 pairs of 101 base-pair (bp) long sequence reads (Table 1). After removing low-quality sequences, 25,017,948 clean pair-end sequence reads remained (Table 1). All these clean reads were assembled into loci (see Materials and Methods). Retaining the longest transcript of each locus yielded 67,683 distinct uni-genes. The minimum length of these uni-genes was 100 bp, the maximum length was 50,138 bp, and the N_50_ was 1675 bp (Table 1). The size distribution indicated that 9893/67,683 uni-genes were longer than 1000 bp (Figure 1).

To construct our phylogenomic data matrix, we used 22 holometabolous transcriptomes; 14 of these transcriptomes were from taxa belonging to the Neuropterida, and constitute the ingroup, whereas the remaining 8 represent the 8 other orders of Holometabola and were used as outgroups (Table S1, Materials and Methods). Orthologs of 2675 pre-selected Benchmarking Universal Single-Copy Orthologs (BUSCO) [29] genes that are conserved and broadly single-copy in arthropods were identified from the 22 transcriptomes. We retrieved 1392 orthologous genes that are single-copy and present in more than half of the 22 transcriptomes, resulting in a phylogenomic data matrix that contained 1,666,191 nucleotide (nt) sites and a translated amino acid (aa) version of the data matrix that contained 555,397 sites.

For each of the nt and aa versions of the data matrix, we also constructed several sub-datasets on the basis of the average bootstrap support (ABS) or relative tree certainty all (RTCA) values of the individual gene trees (see Materials and Methods).

### 2.2. Phylogenetic Analysis Under a Site-Homogeneous Model

**Figure 2:**
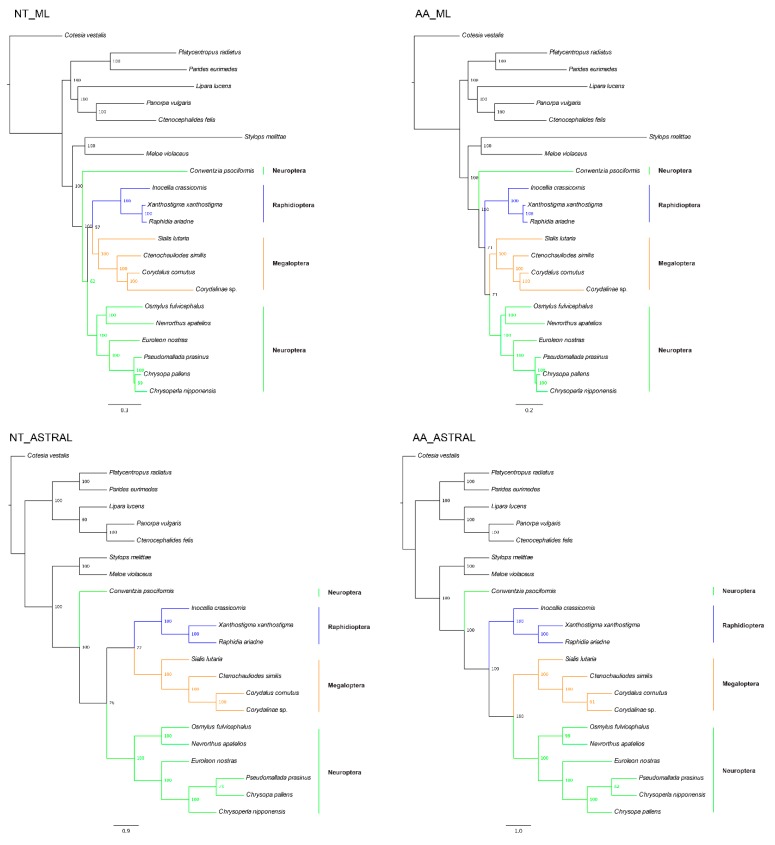
Phylogenetic reconstructions of Neuropterida using different dataset and different methods under the site-homogeneous model. The numbers on the right of each node are the bootstrap support values. Branch color represents the different order of Neuropterida (Blue for Raphidioptera, orange for Megaloptera, and green for Neuroptera). NT: Nucleotide. AA: Amino acid. ML: The concatenated tree from maximum likelihood. ASTRAL: The coalescent tree from ASTRAL.

Both concatenation and species coalescence analyses of the nucleotide (nt) and amino acid (aa) data matrices using a site-homogeneous model recovered a monophyletic Neuropterida. Within Neuropterida, analyses of aa and nt data matrices recovered different topologies (Figure 2). Specifically, analyses of the aa data matrix recovered Megaloptera as the sister group to Neuroptera in both concatenation (maximum likelihood (ML), bootstrap support (BS) = 57) and species coalescence approaches (BS = 77). In contrast, analyses of the nt data matrix recovered Megaloptera as the sister group to Raphidioptera in both concatenation (ML, BS = 71) and species coalescence approaches (BS = 100). Both Megaloptera and Raphidioptera were recovered as monophyletic lineages. In all analyses, the coniopterygid species *Conwentzia psociformis* were identified as the basal or earliest diverging branch of the superorder Neuropterida, suggesting that the order Neuroptera is paraphyletic.

### 2.3. Phylogenetic Analysis Using Genes with Strong Signals

**Figure 3:**
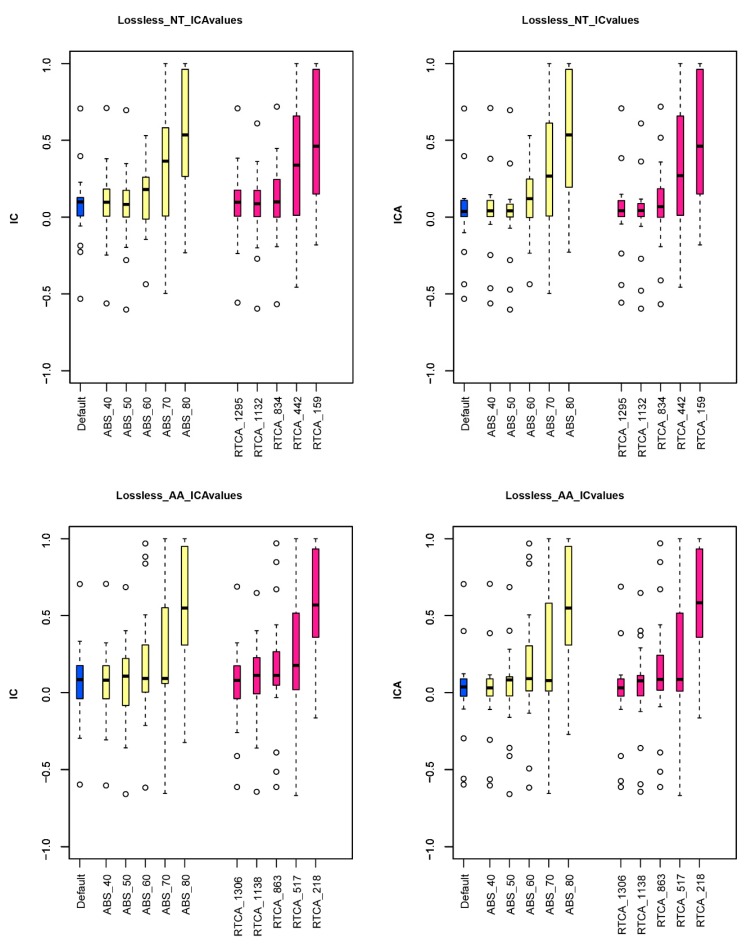
The changes of internode certainty (IC) values as well as internode certainty including all conflicting bipartitions (ICA) values of nucleotide (NT) as well as amino acid (AA). Blue represents the default analysis, yellow represents using genes with different average bootstrap support (ABS), and pink represents using genes with different relative tree certainty including all conflicting bipartitions (RTCA). The filter increases from left to right in every color.

To test whether using genes with stronger phylogenetic signals can reduce incongruence, we examined the phylogenetic behavior of different subsets of genes in the aa and nt data matrices. For the nt data matrix, we performed analyses on five different data matrices comprising genes whose maximum likelihood (ML) trees had average bootstrap support (ABS) values across all internodes greater than or equal to 40% (1295 genes), 50% (1132 genes), 60% (834 genes), 70% (442 genes), or 80% (159 genes), as well as five data matrices comprising the 1295, 1132, 834, 442, or 159 genes whose ML trees had the highest relative tree certainty including all conflicting bipartitions (RTCA) values. For the aa data matrix, we performed analyses on five different data matrices comprising genes whose ML trees had ABS values across all internodes greater than or equal to 40% (1306 genes), 50% (1138 genes), 60% (863 genes), 70% (517 genes), 80% (218 genes), or 87% (72 genes), as well as five data matrices comprising the 1295, 1132, 834, 442, 159, or 65 genes whose ML trees had the highest RTCA values. Gene selection was solely based on the strength of phylogenetic signals exhibited in their gene trees (measured by ABS or RTCA) without any consideration to the topology supported. Each of these data matrices were analyzed using both concatenation and species coalescence approaches. In all cases, both the internode certainty (IC) and the internode certainty including all conflicting bipartitions (ICA) values of the vast majority of internodes greatly increased in data matrices comprised of genes with higher ABS or RTCA values (Figure 3, Table 2 and Table 3), suggesting that selecting genes with high ABS or high RTCA significantly reduced incongruence in the Neuropteridan phylogeny (Figure 3, Table 2; Table 3).

**Figure 4:**
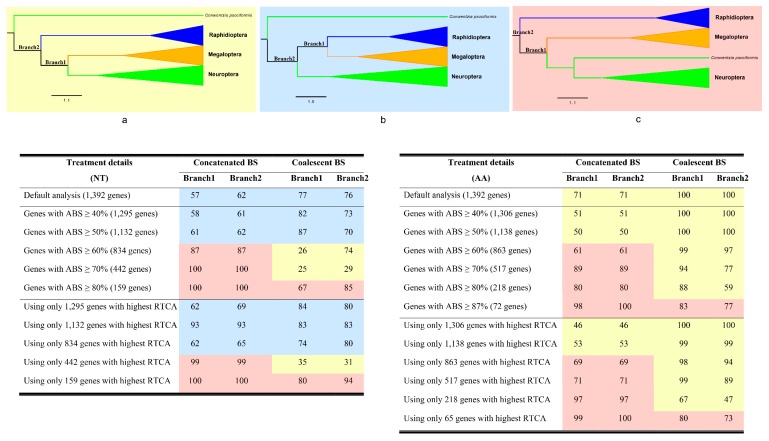
Topology changes as using genes with strong phylogenetic signals under homogeneous model. Different background color indicates the different topology, respectively. Yellow: Topology (**a**); blue: Topology (**b**); pink: Topology (**c**). NT: Nucleotide. AA: Amino acid. ABS: average bootstrap support. RTCA: Relative tree certainty including all conflicting bipartitions.

Examination of the phylogenies of data matrices comprised of genes with higher ABS or RTCA values showed that most relationships were consistent with those inferred from the original data matrix. The main difference was the placement of *C. psociformis* (Neuroptera: Coniopterygidae) (Figure 4). In general, analyses of data matrices that used low stringency filters (e.g., ABS ≥ 40%) placed *C. psociformis* as the basal branch of Neuropterida with either Megaloptera being the sister group to Neuroptera (in aa data matrices) or to Raphidioptera (in nt data matrices). In contrast, analyses of data matrices that used high stringency filters (e.g., ABS ≥ 80%) placed *C. psociformis* as the basal branch of Neuroptera and recovered the order as monophyletic. In this topology, Megaloptera was recovered as the sister group to Neuroptera and the two orders together were the sister group to Raphidioptera (Figure 4).

### 2.4. Heterogeneous Sequence Divergence Test

**Figure 5:**
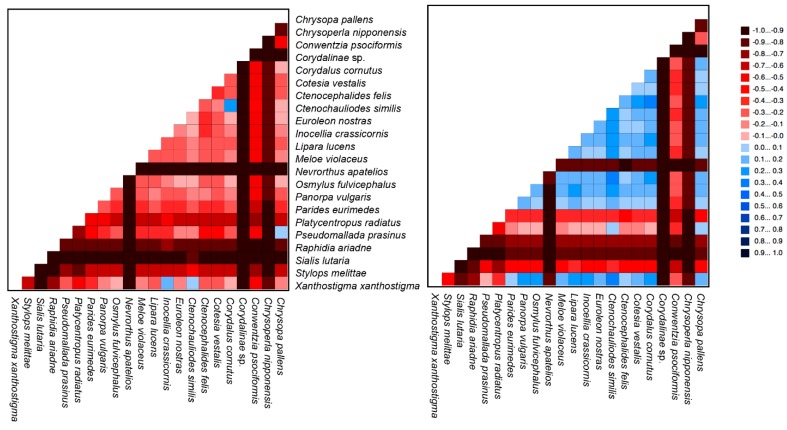
AliGROOVE analysis for nucleotide (NT) and amino acid (AA) sequences. The mean similarity score between sequences is represented by a colored square, based on AliGROOVE scores from -1, indicating great differences in rates from the remainder of the dataset, i.e., heterogeneity (red), to +1, indicating rates match all other comparisons (blue).

Recent phylogenetic studies of arthropods have increasingly shown that heterogeneous models could perform better than homogeneous models in resolving ancient relationships, which are often susceptible to systematic errors such as long branch attraction [30,31,32]. These studies have indicated that homogenous models are unable to accommodate the among-site or among-branch variations in evolutionary patterns such as rate, base composition, and substitution profile (e.g., [9,33]). To test whether there is such heterogeneity in the data and if heterogeneous models need to be used for the phylogenetic reconstruction, we next used the AliGROOVE [34] procedure to test the extent of sequence similarity and alignment ambiguity in pairwise sequence comparisons derived from the nt and aa data matrices. This analysis found strong heterogeneity in sequence divergence for both data matrices (Figure 5). In particular, pairwise sequence comparisons of nt data yielded extremely low scores in almost all species, while pairwise sequence comparisons of aa data received relatively higher scores (Figure 5).

### 2.5. Phylogenetic Analysis Using Site-Heterogeneous Model

**Figure 6:**
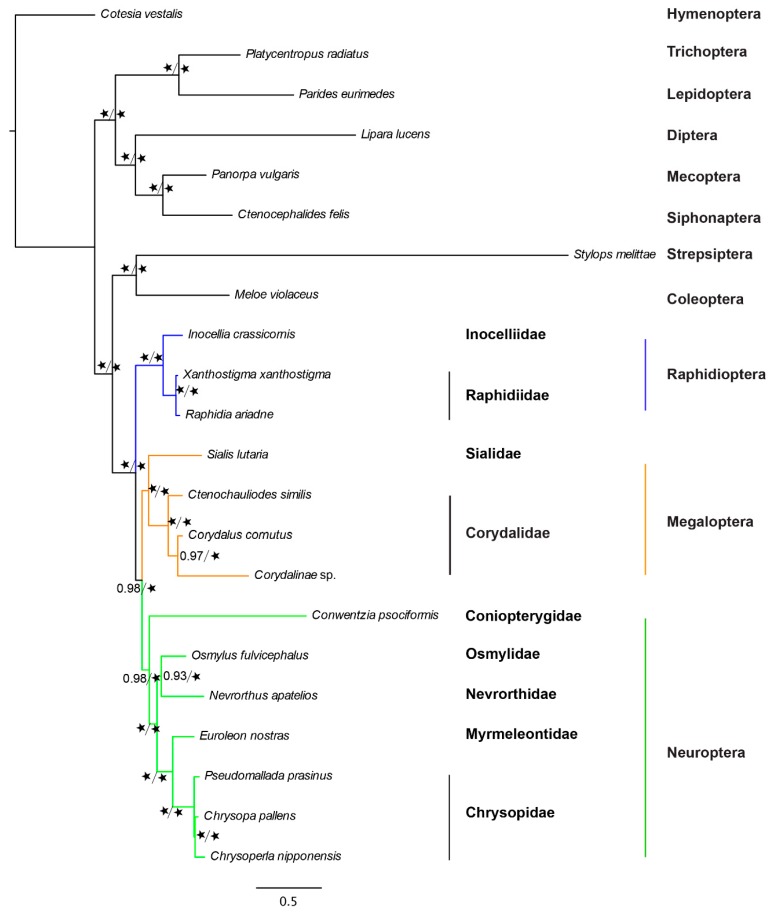
Phylogenetic reconstructions of Neuropterida based on amino acid sequences under site- heterogenous models. The data included all 1392 orthologous genes. The same topology was recovered by both Bayesian analysis under the CAT-Poisson model in Phylobayes and maximum-likelihood analysis under the LG+C60+F model in the IQ-TREE phylogenetic inference software. The two support values shown for each branch are Phylobayes posterior probability (**left**) and IQ-TREE ultrafast bootstrap support (**right**). The black asterisk indicates maximum support values.

Analyses of the entire aa data matrix using the CAT—Poisson site-heterogeneous model in PhyloBayes v4.1c [35] and the LG+C60+F mixture model [36] in IQ-TREE [37] both recovered the same topology as the analysis of nt and aa data matrices that used high stringency filters (Figure 6). Neuropterida was recovered to be monophyletic. Within Neuropterida, the sister-group relationship between Megaloptera and Neuroptera was recovered with high support (posterior probability (pp) = 0.98 and ultrafast bootstrap support (UFBS) = 100%). Megaloptera was recovered to be monophyletic with absolute support (pp = 1 and UFBS = 100%) and the two subfamilies Corydalinae and Chauliodinae, traditionally placed within the family Corydalidae, were grouped as monophyletic (pp = 1 and UFBS = 100%). Coniopterygidae was recovered as the basal branch in Neuroptera (pp = 0.98 and UFBS = 100%), Nevrorthidae as the sister group to Osmylidae (pp = 0.93 and UFBS = 100%), and Myrmeleontidae as the sister group to Chrysopidae (pp = 1 and UFBS = 100%).

## 3. Discussion

The dramatically decreased cost of the whole-genome and transcriptome sequencing has facilitated the generation of genome-scale data from a wide variety of organisms. For insects, there are at least 138 whole genomes and 116 transcriptomes currently available [38]. These large datasets undoubtedly provide significant molecular evidence toward the understanding of the phylogeny and evolution of insects. However, figuring out how to properly use such large amounts of data to reconstruct the insect phylogeny is challenging.

By far, most published insect phylogenies based on genomic or transcriptomic data have been inferred using the concatenation approach on the entire data matrix, without filtering any orthologs that may lack phylogenetic signal [2,21,39,40,41]. Analyses based on concatenation of all orthologous genes in a data matrix almost always results in absolute support values for most internodes of a phylogeny [2,42]. However, absolute support values do not necessarily indicate the reliability of a phylogeny [27,43]. Several case studies have shown that most individual gene trees in phylogenomic studies are topologically incongruent with each other and with the phylogeny supported from concatenation [23,27,29,40,42,44].

Incongruences are prevalent in the phylogenetic analyses and might be caused by both biological and analytical factors. Biological factors such as gene duplication and loss, recombination, natural selection, horizontal gene transfer, as well as incomplete lineage sorting (ILS) [45,46,47,48] can result in genuine differences between the evolutionary histories of genes and species, and some common solutions include careful gene selection (e.g., to avoid paralogy or horizontal gene transfer) and specialized phylogenetic approaches (e.g., coalescent methods for ILS). On the other hand, analytical factors such as stochastic error (e.g., insufficient taxon samples or sequence length) or systematic error (improper model assumptions) can introduce errors into the phylogenetic reconstruction, and might be potentially reduced by the increased sampling of genes and/or taxa, and with some data filtering approaches, such as using genes with high phylogenetic information content [40], slowly evolving genes [49], genes with stationary base composition [50], and so on.

In this study, we mainly investigated the impact of selecting genes that are highly informative or phylogenetic models that are more realistic on the reconstruction of Neuropterida phylogeny. Our results showed that the monophyly of Raphidioptera, Megaloptera, and Neuropterida (Raphidioptera + (Neuroptera + Megaloptera)) were consistently recovered as monophyletic clades, whereas the monophyly of Neuroptera was obtained only if genes with strong signals were analyzed or models that are more realistic were applied. It has been recently shown that phylogenomic data sets may contain genes that are highly informative but yet highly biased. In other words, some genes may have well supported phylogenies that are different from the underlying species tree, and they may bias the phylogenetic reconstruction under our gene selection criterion. Importantly, here the same topology was recovered by both data filtering and model selection, two independent strategies to improve phylogenetic inference, suggesting that our results were unlikely to be dominated by a few strongly biased genes. In addition, the monophyly of Neuropterida and each of the three orders are consistent with several recent phylogenetic studies based on the mitochondrial genome or transcriptome data [2,9,10,21,51].

Within Megaloptera, Corydalinae was recovered as the sister group of Chauliodinae through all the analytical methods, supporting the traditional monophyletic Corydalidae. Coniopterygidae was recovered as the basal branch in Neuroptera, which is consistent with Withycombe and Misof et al. [2,13], as well as Wang et al. [51]. Osmylidae was recovered as the sister group to Nevrorthidae, which is consistent with Winterton et al. [12], which was recovered as a sister group to the rest of Neuroptera with the exclusion of Coniopterygidae, Nevrorthidae, and Sisyridae based on the complete mitochondrial genome [51]. Besides, Gillung et al. [52] reported that NT data gave the better result because AA models were inadequate. However, the NT results with less filtering genes gave incomprehensible topology (Raphidioptera being sister group to Megaloptera), while the AA data gave the better one in this study. Furthermore, our results clearly show that using genes with stronger phylogenetic signals could significantly reduce the incongruence between different datasets as well as between different methods of phylogenetic inference.

Our study presented a comparison between the concatenated method and coalescent method using different datasets under the site-homogeneous and site-heterogeneous model in a transcriptome phylogenomic analysis of Neuropterida insects. Interestingly, analyses using genes with stronger phylogenetic signals under the site-homogeneous model from either concatenated or coalescence approaches and analysis of the AA data matrix under the site-heterogeneous model, yielded identical topologies, in which Neuroptera were recovered as monophyletic. In contrast, inclusion of genes with low phylogenetic signal under the site-homogeneous model in both concatenation and coalescence analyses yielded a paraphyletic Neuroptera. These results suggest that both selections of genes with strong phylogenetic signals as well as the use of more realistic models of sequence evolution are likely to be important in efforts to reconstruct a more accurate tree of insects. Meanwhile, in order to decrease the large computational resources and time, using genes with stronger phylogenetic signals may have a broader prospect as an efficient and accurate approach in the phylogenomic studies of insects.

## 4. Materials and Methods

### 4.1. Insect Samples and RNA Extraction

The *C. similis* specimen used in this experiment was collected from Daming Mount, Guangxi Province, China, on 12 May 2014. To obtain as many gene transcripts as possible, the whole body was sampled and frozen immediately in liquid nitrogen, and stored at −80 °C. Total RNA was extracted using the TRIzol reagent (Invitrogen, Carlsbad, CA, USA) following the manufacturer’s protocol. RNA contamination and degradation were monitored on 1% agarose gels. Other quality parameters, such as purity, concentration, and integrity, were examined using the NanoPhotometer^®^ spectrophotometer (IMPLEN, CA, USA), the Qubit^®^ RNA Assay Kit in Qubit^®^2.0 Fluorometer (Life Technologies, Carlsbad, CA, USA), and the RNA Nano 6000 Assay Kit of the Agilent Bioanalyzer 2100 system (Agilent Technologies, Santa Clara, CA, USA).

### 4.2. cDNA Library Construction and Sequencing

Illumina sequencing was completed by Biomarker Technologies (Beijing, China), with the use of an Illumina HiSeq™ 2500. The first-strand cDNA was synthesized using random hexamer-primers from purified Poly (A) mRNA. Second-strand cDNA was synthesized using buffer, dNTPs, RNaseH and DNA polymerase I. Short fragments were purified using a QiaQuick PCR extraction kit. These fragments were washed with ethidium bromide (EB) buffer for end reparation poly (A) addition and then ligated to sequencing adapters. Suitable fragments, as judged by agarose gel electrophoresis, were selected for use as templates for PCR amplification. The cDNA library was sequenced on Illumina HiSeq™ 2500 using paired-end technology with a 101 base-pair long read in a single run.

### 4.3. Transcriptome Analysis and Assembly

The 26,988,698 raw sequence reads were first filtered to remove poor quality reads using Trimmomatic v0.32 [53] with the following parameters “ILLUMINACLIP: TruSeq3-PE.fa:2:30:10 SLIDINGWINDOW:4:5 LEADING:5 TRAILING:5 MINLEN:25”. After this filtering, 25,017,948 pair-end reads remained. Then, transcriptome de novo assembly was carried out with SOAPdenovo-Trans v1.03 (k-mer size = 31) [54], and 71,115 transcripts were obtained. The longest transcript of each locus was collected to generate 67,683 uni-genes as the final assembly. All raw transcriptome data have been deposited in the NIH Short Read Archive (SRA) with the accession number SAMN05525730.

### 4.4. Data Matrix Construction

We used the complete sets of annotated orthology data of 22 holometabolous transcriptomes (Table S1). The ingroup taxa include 14 species of Neuropterida, which represent three orders within the superorder and all families with available transcriptomes. The remaining 8 species represent 8 other orders of Holometabola and were selected as outgroups. Almost all the transcriptome data were downloaded from Transcriptome Shotgun Assembly (TSA) database of GeneBank (http://www.ncbi.nlm.nih.gov) with the accession number in the Table S1, except *Chrysopa nipponensis* and the newly sequenced *C. similis*. The transcriptome of *C. nipponensis* was downloaded from the Sequence Read Archive (SRA) of GeneBank. Both of these transcriptomes were assembled using SOAPdenovo-Trans v1.03 [54] since all other transcriptomes from TSA were assembled by SOAPdenovo-Trans [2,21]. Each transcriptome assembly was assessed for the copy number of 2675 pre-selected genes that were single-copy in 38 arthropod genomes in the OrthoDBv7 database using BUSCO v1.1b1 [29,55]. In total, 1392 genes were found to be present in more than 50 percent of the 22 species examined in this study, and their coding sequences and the respective translated amino acid sequences were retrieved to construct the phylogenomic data matrices. A series of different sub-datasets was constructed using custom Perl scripts. ABS and RTCA values were used to construct eight sub-datasets of 1392 orthologs: Five sub-datasets for comprising genes whose ML trees had ABS values across all internodes greater than or equal to 40% (1295 genes), 50% (1132 genes), 60% (834 genes), 70% (442 genes), or 80% (159 genes), and five sub-datasets comprising the 1295, 1132, 834, 442, or 159 genes whose ML trees had the highest RTCA values. For amino acids, we analyzed five sub-datasets comprising genes whose ML trees had ABS values across all internodes greater than or equal to 40% (1306 genes), 50% (1138 genes), 60% (863 genes), 70% (517 genes), 80% (218 genes), or 87% (72 genes), and five sub-datasets comprising the 1295, 1,132, 834, 442, 159, or 65 genes whose ML trees had the highest RTCA values.

### 4.5. Gene Alignment

We aligned all genes using the MAFFT software, v7.182 [56] based on their amino acid sequence, using E-INS-i (mafft—maxiterate 1000—reorder—genafpair). Then, we used PAL2NAL [57] to translate amino acid sequence alignments to codon sequence alignments, and the “gappyout” option of trimAl [58] to trim the amino acid sequence alignments. Trimmed segments of the amino acid sequence alignments were deleted from their corresponding codon sequence alignments using custom Perl scripts. Following trimming, our data matrix consisted of 1392 genes from 22 species.

### 4.6. Phylogenetic Inference Under Site-Homogeneous Model

For the codon sequence and amino acid alignments of each gene, the un-rooted phylogenetic tree under the optimality criterion of maximum likelihood (ML) was inferred using the RAxML, version 8.0.20 [59], under the GTRGAMMA (codon sequence) and PROTGAMMAAUTO (amino acids) model. The values of the nucleotide base/amino acid frequencies were fixed to “observed” and those of the substitution rate parameters estimated from the data. For the concatenation analysis, codon sequence and amino acid alignments from all genes were analyzed as a single super-matrix.

The un-rooted concatenation species phylogeny was inferred through a single ML search in RAxML v8.0.20 [59], with the values of the nucleotide base/amino acid frequencies fixed to “observed” and those of the substitution rate parameters estimated from the data. The concatenated file was partitioned based on every gene, and the model for every nucleotide sequence was GTRGAMMA and the model for every amino acid sequence was extracted from the single gene tree analysis. In all cases, robustness in inference was assessed via bootstrap resampling (100 replicates). Note that the RAxML software first infers the topologies for each of the bootstrap replicates and then searches for the best-scoring ML tree using every fifth bootstrap replicate tree as a starting tree.

The coalescent species phylogeny was estimated using 100 replicates of multi-locus bootstrapping in ASTRAL [24] (java -Xmx36000M -jar astral.4.7.8.jar -i TREECOLLECTION -o OUTPUT -b BS_PATH -r 100).

### 4.7. Phylogenetic Inference Under Site-Heterogeneous Model

Analysis of the entire aa data matrix using the CAT–Poisson site-heterogeneous model (among site variation in stationary frequencies is modeled by a Dirichlet process and exchange rates among amino acids are assumed to be equal) was conducted in PhyloBayes v4.1c [35]. Four independent Markov Chain Monte Carlo (MCMC) chains were run in parallel for at least 2000 cycles until the convergence between the four chains were considered acceptable (the maxdiff parameter below 0.3). A consensus tree was obtained by discarding 25% of the samples as burn-in, and then sampling a tree every 10 cycles from the remaining samples. At the same time, we also analyzed the aa data matrix under the maximum-likelihood framework using the empirical site-heterogeneous model LG+C60+F [36] implemented in IQ-TREE v1.6.9 [37]. Here, the reliability of inferred relationships was assessed via ultrafast bootstrap approximation 2 plus a final nearest-neighbor-interchange based optimization (UFBoot2+NNI) [60] with 1000 replicates.

### 4.8. Evaluation of Incongruence

Internode certainty (IC), internode certainty including all conflicting bipartitions (ICA) and tree certainty including all conflicting bipartitions (TCA), relative tree certainty including all conflicting bipartitions (RTCA) [27,28] were calculated using RAxML v8.0.20 [59] (raxmlHPC-PTHREADS-SSE3 -T 8 -f i -t REFERENCETREE -z TREECOLLECTION -m PROTGAMMAAUTO -n NAME).

### 4.9. Data Availability

All data and analyses described in this study are deposited at Figshare under the accession 10.6084/m9.figshare.3504290.

## Figures and Tables

**Table 1:** Summary of *Ctenochauliodes similis* transcriptome.

Name	Number
Raw Data	26,988,698
Q_20_ of string one of raw data	91.70%
Q_20_ of string two of raw data	89.91%
GC content of string one raw data	40.64%
GC content of string two raw data	40.62%
Total number of clean reads	25,017,948
Q_20_ of string one of clean reads	99.98%
Q_20_ of string two of clean reads	99.95%
GC content of string one of clean reads	40.26%
GC content of string two of clean reads	40.21%
Total number of unigenes	67,683
Minimum length of unigenes	100
Maximum length of unigenes	50,138
Mean length of unigenes	585.26
N_50_ of unigenes (nt)	1675

**Table 2:** Differences in holometabolous phylogenies inferred from different phylogenomic practices for nucleotides.

Treatment (NT)	Treatment Details	TCA	RTCA	ICA Increases	ICA Decreases
Default analysis	1392 genes	1.41	0.07	/	/
Selection of genes whose ML trees have high ABS	Genes with ABS ≥ 40% (1295 genes)	1.45	0.08	8	11
	Genes with ABS ≥ 50% (1132 genes)	1.23	0.07	5	14
	Genes with ABS ≥ 60% (834 genes)	2.51	0.13	14	5
	Genes with ABS ≥ 70% (442 genes)	6.41	0.34	18	1
	Genes with ABS ≥ 80% (159 genes)	10.42	0.55	16	3
Selection of genes whose ML trees have high RTC	Using only 1295 genes with the highest RTC	1.47	0.08	5	10
	Using only 1132 genes with the highest RTC	1.20	0.06	9	10
	Using only 834 genes with the highest RTC	2.05	0.11	11	7
	Using only 442 genes with the highest RTC	6.20	0.33	16	3
	Using only 159 genes with the highest RTC	10.11	0.53	17	2

The specific phylogenomic practice tested (treatment) the tree certainty including all bipartitions (TCA) of the phylogeny, the relative tree certainty including all bipartitions (RTCA) of the phylogeny, the numbers of internodes of the insect phylogeny in which the numbers of internodes of the insect phylogeny in which internode certainty including all bipartitions (ICA) increases or decreases. As the maximum value of ICA for a given internode is 1, the maximum value of TCA for a given phylogeny is the number of internodes, which are 19.

**Table 3:** Differences in holometabolous phylogenies inferred from different phylogenomic practices for amino acids.

Treatment (AA)	Treatment Details	TCA	RTCA	ICA Increases	ICA Decreases
Default analysis	1392 genes	1.13	0.06	/	/
Selection of genes whose ML trees have high ABS	Genes with ABS ≥ 40% (1306 genes)	1.08	0.06	5	14
	Genes with ABS ≥ 50% (1138 genes)	1.33	0.07	11	7
	Genes with ABS ≥ 60% (863 genes)	3.48	0.18	13	6
	Genes with ABS ≥ 70% (517 genes)	4.65	0.24	12	7
	Genes with ABS ≥ 80% (218 genes)	10.38	0.55	17	2
Selection of genes whose ML trees have high RTC	Using only 1306 genes with the highest RTC	0.94	0.05	4	15
	Using only 1138 genes with the highest RTC	1.55	0.08	11	8
	Using only 863 genes with the highest RTC	2.85	0.15	11	8
	Using only 517 genes with the highest RTC	4.73	0.25	14	5
	Using only 218 genes with the highest RTC	11.09	0.58	17	2

The specific phylogenomic practice tested (treatment) the tree certainty including all bipartitions (TCA) of the phylogeny, the relative tree certainty including all bipartitions (RTCA) of the phylogeny, the numbers of internodes of the insect phylogeny in which the numbers of internodes of the insect phylogeny in which internode certainty including all bipartitions (ICA) increases or decreases. As the maximum value of ICA for a given internode is 1, the maximum value of TCA for a given phylogeny is the number of internodes, which are 19.

## Supplementary Materials

Supplementary materials can be found at https://www.mdpi.com/1422-0067/20/5/1072/s1.

## Abbreviations

ABS	Average bootstrap support
nt	Nucleotide
aa	Amino acid
BS	Bootstrap support
UFBS	Ultrafast bootstrap support
ML	Maximum likelihood
IC	Internode certainty
ICA	Internode certainty including all conflicting bipartitions
TC	Tree certainty;
TCA	Tree certainty including all conflicting bipartitions
pp	Posterior probability
RTCA	Relative tree certainty including all conflicting bipartitions;
EB	Ethidium bromide
SRA	Short Read Archive
TSA	Transcriptome Shotgun Assembly
