# Supplementary material for: Gene Selection and Evolutionary Modeling Affect Phylogenomic Inference of Neuropterida Based on Transcriptome Data
Source: Int J Mol Sci. 2019 Mar 1;20(5):1072. doi: 10.3390/ijms20051072 (PMC6429444; doi:10.3390/ijms20051072)
Supplement: Supplementary file 1 [file ijms-20-01072-s001.pdf]

Table S1. Summary of taxonomic groups used in this study with Genbank accession numbers.

Order	Family	Subfamily	Species	Accession Number
**Outgroup:**				
Coleoptera	Meloidae		*Meloe violaceus*	GATA02000000
Diptera	Chloropidae		*Lipara lucens*	GAZD02000000
Hymenoptera	Braconidae		*Cotesia vestalis*	GAUP02000000
Lepidoptera	Papilionidae		*Parides eurimedes*	GAXH02000000
Mecoptera	Panorpidae		*Panorpa vulgaris*	GAUH02000000
Siphonaptera	Pulicidae		*Ctenocephalides felis*	GAYP02000000
Strepsiptera	Stylopidae		*Stylops melittae*	GAZM02000000
Trichoptera	Limnephilidae		*Platycentropus radiatus*	GASS02000000
**Ingroup:**				
Megaloptera	Corydalidae	Corydalinae	*Corydalus cornutus*	GATG02000000
	Corydalidae	Corydalinae	*Corydalinae sp.*	GADH01000000
	Corydalidae	Chauliodinae	*Ctenochauliodes similis*	This study
	Sialidae		*Sialis lutaria*	GABK01000000
Neuroptera	Chrysopidae		*Pseudomallada prasinus*	GAVV02000000
	Chrysopidae		*Chrysopa pallens*	GAGF01000000
	Chrysopidae		*Chrysopa nipponensis*	SRR1653342
	Coniopterygidae		*Conwentzia psociformis*	GAYH02000000
	Myrmeleontidae		*Euroleon nostras*	GAXW02000000
	Nevrorthidae		*Nevrorthus apatelios*	GACU01000000
	Osmylidae		*Osmylus fulvicephalus*	GAYC02000000
Raphidioptera	Inocelliidae		*Inocellia crassicornis*	GAZH02000000
	Raphidiidae		*Xanthostigma xanthostigma*	GAUI02000000
	Raphidiidae		*Raphidia ariadne*	GACX01000000
